# The complete chloroplast genome sequence of *Aeschynanthus acuminatus* Wall. ex A. DC., a Yao medicinal plant in China

**DOI:** 10.1080/23802359.2026.2715817

**Published:** 2026-08-14

**Authors:** Yihan Jiang, Jiacheng Lu, Ze Xu, Fang Wen

**Affiliations:** ^a^ School of Public Health, Wenzhou Medical University, Wenzhou, China; ^b^ The Second School of Medicine, Wenzhou Medical University, Wenzhou, China; ^c^ Guangxi Institute of Botany, Guangxi Zhuang Autonomous Region and Chinese Academy of Sciences, Guilin, China; ^d^ Guangxi Key Laboratory of Plant Functional Phytochemicals and Sustainable Utilization, Guilin, China

**Keywords:** *Aeschynanthus acuminatus*, chloroplast genome, Yao medicine, ethnomedicine

## Abstract

The Yao ethnomedicine "Xiaobai-beifeng" uses *Aeschynanthus acuminatus*, yet its chloroplast genome was uncharacterized. We report the first complete chloroplast genome (153,158 bp), a typical quadripartite structure encoding 125 unique genes. Phylogenomic analysis places A. acuminatus as sister to *Lysionotus*. This study provides a genomic resource for molecular identification and conservation of this medicinal plant.

## Introduction

Yao medicine is an ethnopharmaceutical system developed by the Yao people, an ethnic minority residing in the mountainous regions of southern China. Rooted in local medicinal flora and fauna, it is built upon the “104 Lao-ban-yao” as its foundational materia medica and features distinct diagnostic and therapeutic principles (Yan et al. 2022).

*Aeschynanthus acuminatus* Wall. ex A.DC.(1825) (Xiaobai-beifeng), one of the “Seventy-two Feng” remedies within the Lao-ban-yao tradition, is traditionally employed to calm the mind, dispel wind-dampness, and alleviate swelling and pain (He et al. 2016a). It is indicated for conditions such as edema, epilepsy, rheumatic arthralgia, and bone fractures (He et al. 2016b). According to Flora of China (Wu et al. 2011), *A. acuminatus* is an epiphytic subshrub. Its leaves are opposite, glabrous, oblong to narrowly oblanceolate. The inflorescence bears 1–3 red flowers, stamens are exserted. The capsule with seeds bearing a 1.5–4 mm hair at each end. This species typically grows as an epiphyte on forest margins or rocks in valleys and is distributed in Guangxi, Guangdong, Yunnan, and southern Sichuan. However, *A. acuminatus* (Gesneriaceae) exhibits extensive morphological overlap with many congeners, leading to frequent confusion with *Hoya carnosa* (Apocynaceae) (Dabai-beifeng) in the herbal market. This has resulted in unclear medicinal origins, uneven quality, and substantial constraints on precise clinical application and resource utilization.

Chloroplast DNA (cpDNA) is generally maternally inherited, characterized by a highly conserved structural organization, and exhibits differential evolutionary rates between coding sequences and intergenic regions. (Dobrogojski et al. 2020). Sequence variation in the large single-copy (LSC), small single-copy (SSC), and inverted repeat (IR) regions provides high-resolution molecular markers for species delimitation, population genetics, and resource traceability (Daniell et al. 2016).

In this study, we present the first comprehensive assembly, annotation, and structural characterization of the complete chloroplast genome of *A. acuminatus*. These data provide a robust genomic basis for distinguishing closely related congeners and selecting potential DNA barcodes. The plastome resource generated herein supports reliable molecular authentication of *A. acuminatus* and offers a valuable reference for evaluating population genetic diversity and verifying the authenticity of medicinal materials.

## Materials and methods

**Figure 1.**
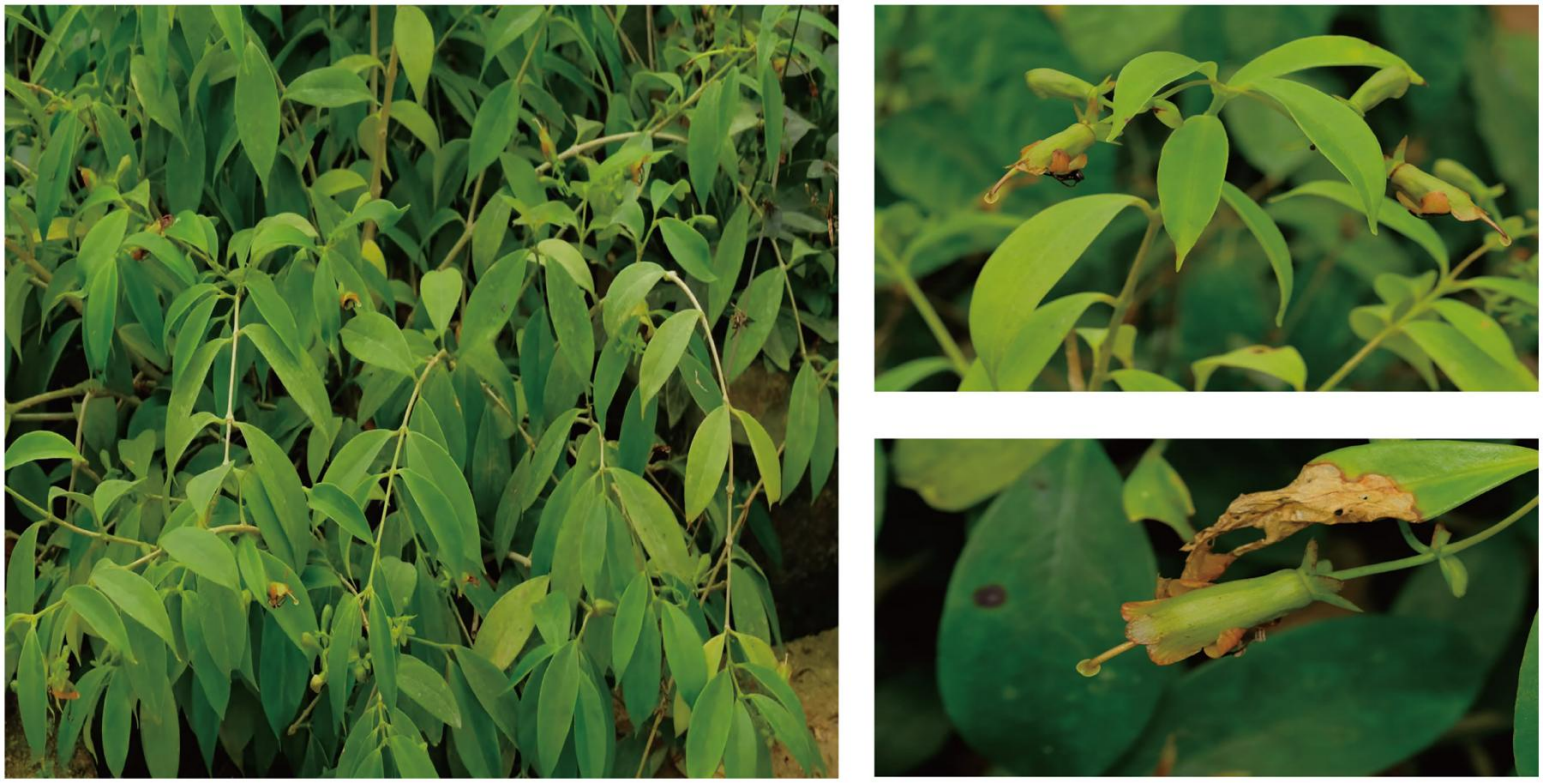
Morphological features of *aeschynanthus acuminatus* cultivated in the medicinal plant garden of Guangxi Institute of Botany, Guilin, China. (A) Overall habit of the plant showing the growth form and leaf arrangement. (B) Close-up view of flowers and leaves, showing the floral structure. (C) Detailed view of a single flower, highlighting the corolla and stamens. Photographs were taken by fang wen and Longfei Fu (35057143@qq.com).

Fresh, healthy leaves of *A. acuminatus* (Figure 1) were collected from cultivated plants maintained at the Guangxi Institute of Botany, Chinese Academy of Sciences (25°01′N, 110°17′E). A voucher specimen (CSF250927) was deposited in the herbarium of the same institute (contact person: Longfei Fu, email: 35057143@qq.com). Total genomic DNA was isolated from 100 mg silica-gel-dried leaf tissue using the standard cetyl-trimethyl-ammonium bromide (CTAB) method (Huang et al. 2000) with 1 % (w/v) polyvinylpyrrolidone (PVP-40) to reduce polyphenol oxidation. DNA quality was checked on 1 % agarose gels, and purity/quantity were assessed with a NanoDrop One spectrophotometer (Thermo Fisher Scientific, USA) and Qubit dsDNA HS Assay (Invitrogen, USA). Only samples with A260/280 ≈ 1.8–2.0 and concentration ≥ 30 ng μL^−1^ were retained.

A paired-end sequencing library with an approximate insert size of 350 bp was prepared using the NEBNex Ultra II DNA Library Prep Kit (New England Biolabs, USA) in accordance with the manufacturer’s instructions. The library was subsequently sequenced on the Illumina NovaSeq 6000 platform (Illumina, USA) at GLBIZZIA BIOSCIENCES CO., LTD. (Beijing), generating 671 Mb of raw sequencing data.

Adapter sequences and low-quality reads (*Q* < 20) were removed with fastp v0.23 (Chen et al. 2018). De novo assembly was performed with GetOrganelle v1.7.6 (Jin et al. 2020) under default k-mer settings (*k* = 21, 39, 65, 85, 105, 127). The final circular consensus was obtained by connecting the two single-copy regions after manual confirmation of the IR boundaries. Coverage depth was > 300× across the entire genome (visualized with R software; Figure S1).

Protein-coding genes, tRNAs and rRNAs were annotated using the dual pipeline CPGAVAS2 (Shi et al. 2019) and GeSeq (Tillich et al. 2017). The circular plastome diagram, cis-splicing gene map and trans-splicing gene map were drawn with the online CPGView server (Liu et al. 2023) and refined in Adobe Illustrator 2022.

Complete chloroplast genomes of 20 Trib. Trichosporeae species, 20 Trib. Didymocarpeae species and one outgroup species (*Plantago strictissima*) were downloaded from NCBI. Ninety-five common protein-coding genes were extracted and aligned individually with MAFFT v7.490 (Katoh & Standley 2013) using the auto strategy, which automatically selects the most appropriate algorithm (L-INS-i for <200 sequences or FFT-NS-2 for ≥200 sequences) based on the input data size. Alignments were concatenated into a supermatrix in PhyloSuite v1.2.3 (Zhang et al. 2020). Maximum-likelihood (ML) phylogeny was inferred with IQ-TREE v2.2.0 (Minh et al. 2020) under the best-fit model TVM+F + I + R4 (TVM: Transversion Model; +F: empirical base frequencies; +I: invariable sites; +R4: four-category free-rate model for among-site rate heterogeneity) selected by ModelFinder (Kalyaanamoorthy et al. 2017), with 1,000 standard bootstrap replicates. Trees were visualized and edited in ITOL (Letunic and Bork 2024).

## Results

**Figure 2.**
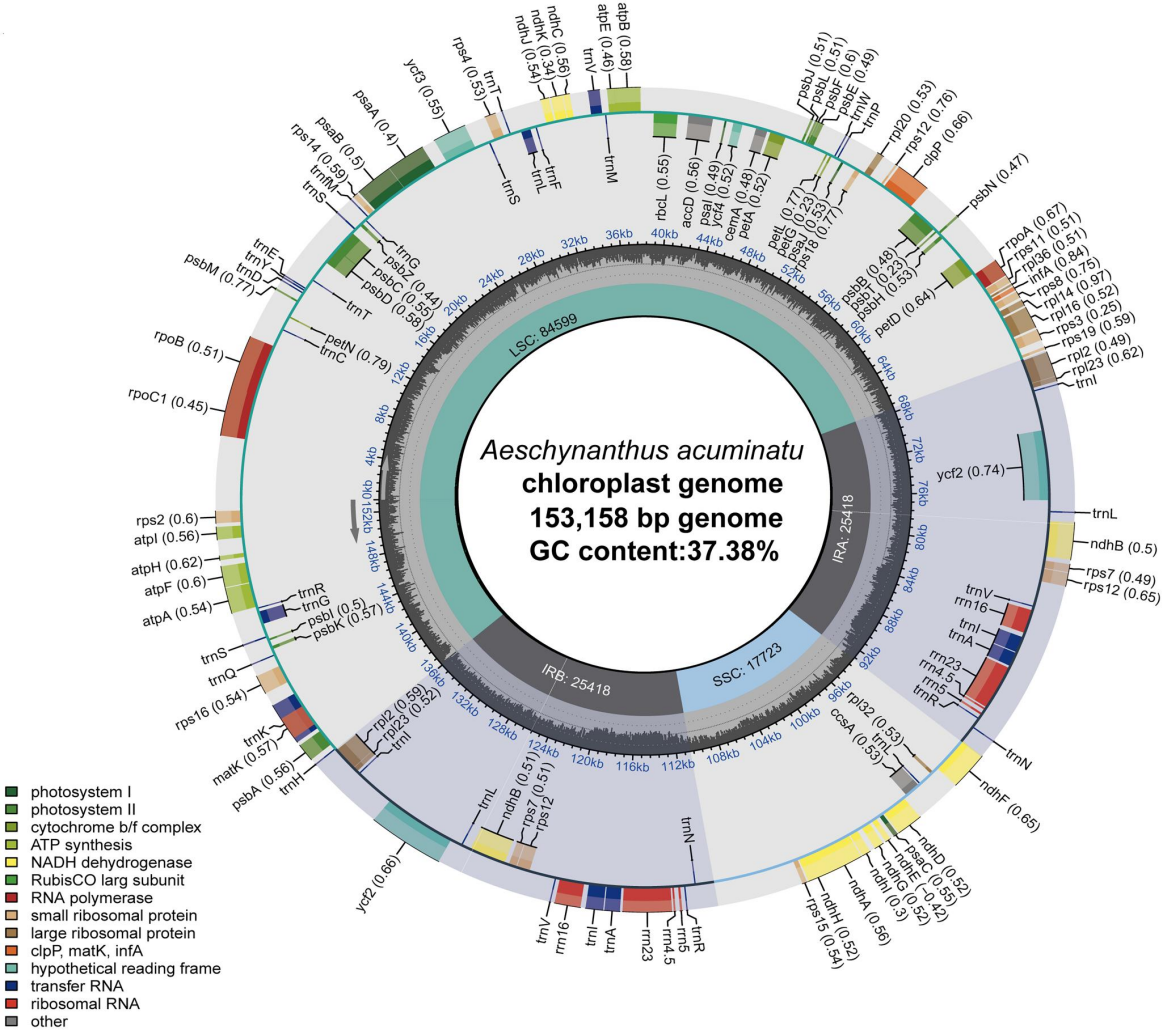
Circular map of the *aeschynanthus acuminatus* chloroplast genome, generated by OGDRAW. From the outside inward, the first circle shows transcription directions of genes; genes on the inside and outside are transcribed clockwise and counter-clockwise, respectively. The different functional groups are indicated by color-code at the left bottom: photosynthesis (green), genetic information processing (red), DNA replication and repair (yellow), and other functions (blue). The numbers on the outer second circle indicate positions in the chloroplast genome. The dark gray parts in the inner second circle show GC content with a cutoff of 50% (dotted line). The third circle represents the four parts of the chloroplast genome, consisting of LSC (large single-copy, green part), SSC (small single-copy, blue part), IRa and IRb (inverted repeat regions, gray part). The genome is 153,158 bp in length (GenBank accession number: PX741072). Further details are provided on the OGDRAW homepage.

The complete chloroplast genome of *A. acuminatus* is 153,158 bp in length (Figure 2). It exhibits the typical quadripartite organization characteristic of angiosperms, consisting of a large single-copy (LSC) region of 84,599 bp, a small single-copy (SSC) region of 17,723 bp, and two inverted repeat (IR) regions (IRa and IRb), each 25,418 bp in length. The GC content is unevenly distributed, with 35.20% in the LSC region, 31.02% in the SSC region, and 43.22% in the IR regions. In total, 125 unique genes were annotated, comprising 80 protein-coding genes (PCGs), 37 tRNA genes, and 8 rRNA genes. A total of 17 genes are duplicated in the IR regions, including six PCGs (*ndhB*, *rpl*2, *rpl*23, *rps*7, *rps*12, *ycf*2), four rRNAs (*rrn*4.5, *rrn*5, *rrn*16, *rrn*23), and seven tRNAs (*trnA-UGC*, *trnI-CAU*, *trnI-GAU*, *trnL-CAA*, *trnN-GUU*, *trnR-ACG*, *trnV-GAC*).

Ten genes contain one cis-spliced intron (*rpoC*1, *petD*, *rpl*16, *rpl*2(2), *ndhB*(2), *ndhA*, *rps*16, *atpF*) and two genes harbor two introns each: *ycf*3 (exon lengths 224-230-125 bp) and *clpP* (exon lengths 225-293-70 bp) (Figure S2). The trans-splicing gene *rps*12 is partitioned into three exons: a 5′ exon located in LSC and two 3′ exons duplicated in IRs (Figure S3).

**Figure 3.**
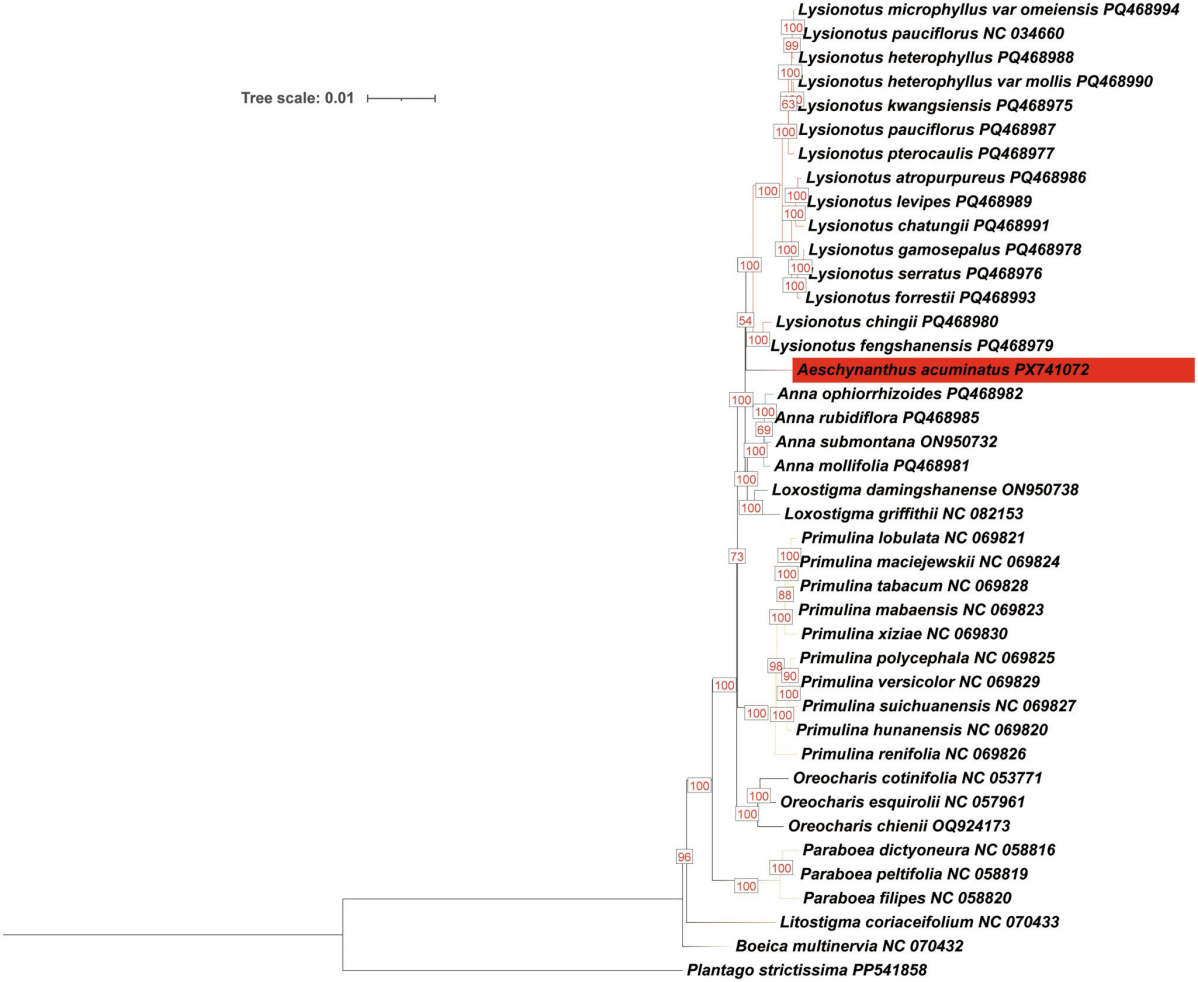
Maximum-likelihood phylogeny of aeschynanthus acuminatus and related genera in gesneriaceae based on 95 concatenated protein-coding genes. The tree was constructed using IQ-TREE under the TVM+F + I + R4 model with 1,000 ultrafast bootstrap replicates. The following sequences were used: *Anna submontane* (ON950732), *A. rubidiflora* (PQ468985), *A. ophiorrhizoides* (PQ468982), *A. mollifolia* (PQ468981), *Loxostigma griffithii* (NC 082153), *L. damingshanense* (ON950738), *Lysionotus pauciflorus* (NC 034660) (Ren et al. 2017), *L. microphyllus* var. *omeiensis* (PQ468994), *L. forrestii* (PQ468993), *L. chatungii* (PQ468991), *L. heterophyllus* var. *mollis* (PQ468990), *L. levipes* (PQ468989), *L. heterophyllus* (PQ468988), *L. pauciflorus* (PQ468987), *L. atropurpureus* (PQ468986), *L. chingii* (PQ468980), *L. fengshanensis* (PQ468979), *L. gamosepalus* (PQ468978), *L. pterocaulis* (PQ468977), *L. serratus* (PQ468976), *L. kwangsiensis* (PQ468975), *Litostigma coriaceifolium* (NC 070433), P*rimulina polycephala* (NC 069825), *P. versicolor* (NC 069829), *P. suichuanensis* (NC 069827), *P. hunanensis* (NC 069820), *P. renifolia* (NC 069826), *P. mabaensis* (NC 069823), *P. xiziae* (NC 069830), *P. tabacum* (NC 069828), *P. lobulata* (NC 069821), *P. maciejewskii* (NC 069824), *Oreocharis chienii* (OQ924173), *O. esquirolii* (NC 057961) (Gu et al. 2020), *O. cotinifolia* (NC 053771) (Tang et al. 2021), P*araboea dictyoneura* (NC 058816), *P. peltifolia* (NC 058819), *P. filipes* (NC 058820), *Haberlea rhodopensis* (NC 058820), *Boeica multinervia* (NC 070432) (Wen et al. 2022), *Plantago strictissima* (PP541858) (outgroup).

Maximum-likelihood (ML) analyses of 95 concatenated PCGs from 20 Trib. Trichosporeae, 20 Trib. Didymocarpeae species, and one outgroup species (*Plantago strictissima*) consistently placed *A. acuminatus* as most closely related to species of the genus *Lysionotus*, with which it forms a well-supported monophyletic group. *A. acuminatus* is more distantly sister to species of the genera *Anna* and *Loxostigma* (Figure 3). All *Lysionotus* accessions were recovered as a successive grade basal to the *Anna* + *Loxostigma* + *Aeschynanthus* assemblage.

Maximum-likelihood (ML) analyses of 95 concatenated PCGs from 20 Trib. Trichosporeae 20 Trib. Didymocarpeae species and one outgroup species (*Plantago strictissima*) consistently place *A. acuminatus* formed an isolated terminal branch that is sister to a maximally supported clade uniting the genera *Anna* and *Loxostigm* (Figure 3). All *Lysionotus* accessions were recovered as a successive grade basal to the *Anna* + *Loxostigma* + *Aeschynanthus* assemblage.

## Discussion and conclusions

This study reports the first complete chloroplast genome assembly of *A. acuminatus*, an ethnomedicinally and culturally significant plant in Yao traditional medicine. The plastome exhibits the canonical quadripartite structure, comprising the LSC, SSC, and two IR regions, and its genome size, GC content, and gene composition are broadly consistent with those documented for most angiosperms (Gao et al. 2023; Mohd Talkah et al. 2024; Sato et al. 1999). These findings agree with plastome structural conservation previously reported in other medicinal plant lineages (Huang & Wee 2020).

Phylogenetic reconstruction based on 95 shared protein-coding genes provided a well-resolved and strongly supported topology within tribe Trichosporeae. *A. acuminatus* is most closely related to species of the genus *Lysionotus*, with which it forms a well-supported monophyletic group, and is more distantly sister to species of the genera *Anna* and *Loxostigma* (Bai et al. 2012; Yang et al. 2025). Our results demonstrate that complete chloroplast genome datasets significantly enhance phylogenetic resolution compared with traditional single-locus markers, particularly in taxonomically complex genera (Daniell et al. 2021).

The generation of this plastome resource holds considerable practical significance. Morphological similarity and frequent synonymy within *Aeschynanthus* have long complicated species delimitation. The complete chloroplast genome provides a reliable molecular barcode that can support precise species authentication, improve the quality control of traditional medicinal materials, and reduce misidentification risk in ethnopharmacological applications (Niu et al. 2023).

In conclusion, this study successfully sequenced, assembled, and annotated the first complete chloroplast genome of *A. acuminatus*. The genome exhibits conserved structural features characteristic of angiosperm plastomes and demonstrates a largely stable gene organization within Gesneriaceae. Future investigations incorporating additional *Aeschynanthus* plastomes and broader taxonomic sampling will further resolve evolutionary relationships and provide deeper insights into plastome diversification within this medicinally important lineage.

## Supplementary Material

Supplemental Material

Supplemental Material

Supplemental Material

## Data Availability

The genome sequence data that support the findings of this study are openly available in GenBank of NCBI at (https://www.ncbi.nlm.nih.gov) under the accession no. PX741072. The associated BioProject, BioSample and SRA, numbers are PRJNA1391071, SAMN54213069, and SRR36527775, respectively.
